# 3D-Printed Micro-Tweezers with a Compliant Mechanism Designed Using Topology Optimization

**DOI:** 10.3390/mi12050579

**Published:** 2021-05-19

**Authors:** Yukihito Moritoki, Taichi Furukawa, Jinyi Sun, Minoru Yokoyama, Tomoyuki Shimono, Takayuki Yamada, Shinji Nishiwaki, Tatsuto Kageyama, Junji Fukuda, Masaru Mukai, Shoji Maruo

**Affiliations:** 1 Graduate School of Engineering Science, Yokohama National University, 79-5 Tokiwadai, Hodogaya-ku, Yokohama 240-8501, Japan; moritoki-yukihito-zm@ynu.jp (Y.M.); sun-jinyi-yc@ynu.jp (J.S.); minoru.yokoyama.35@gmail.com (M.Y.); 2 Faculty of Engineering, Yokohama National University, 79-5 Tokiwadai, Hodogaya-ku, Yokohama 240-8501, Japan; furukawa-taichi-xp@ynu.ac.jp (T.F.); shimono-tomoyuki-hc@ynu.ac.jp (T.S.); kageyama-tatsuto-tp@ynu.ac.jp (T.K.); fukuda@ynu.ac.jp (J.F.); mukai-masaru-vw@ynu.ac.jp (M.M.); 3 Department of Strategic Studies, Institute of Engineering Innovation, School of Engineering, the University of Tokyo, 2-11-16 Yayoi, Bunkyo–ku, Tokyo 113-8656, Japan; t.yamada@mech.t.u-tokyo.ac.jp; 4 Department of Mechanical Engineering and Science, Kyoto University, C3 Kyotodaigaku-katsura, Nishikyo-ku, Kyoto, Kyoto 615-8540, Japan; shinji@prec.kyoto-u.ac.jp; 5 Kanagawa Institute of Industrial Science and Technology (KISTEC), 3-2-1 Sakado Takatsu-ku, Kawasaki, Kanagawa 213-0012, Japan

**Keywords:** topology optimization, 3D printing, microstereolithography, compliant mechanism, micro-manipulator, micro-tweezers

## Abstract

The development of handling technology for microscopic biological samples such as cells and spheroids has been required for the advancement of regenerative medicine and tissue engineering. In this study, we developed micro-tweezers with a compliant mechanism to manipulate organoids. The proposed method combines high-resolution microstereolithography that uses a blue laser and topology optimization for shape optimization of micro-tweezers. An actuation system was constructed using a linear motor stage with a force control system to operate the micro-tweezers. The deformation of the topology-optimized micro-tweezers was examined analytically and experimentally. The results verified that the displacement of the tweezer tip was proportional to the applied load; furthermore, the displacement was sufficient to grasp biological samples with an approximate diameter of several hundred micrometers. We experimentally demonstrated the manipulation of an organoid with a diameter of approximately 360 µm using the proposed micro-tweezers. Thus, combining microstereolithography and topology optimization to fabricate micro-tweezers can be potentially used in modifying tools capable of handling various biological samples.

## 1. Introduction

Microfabrication techniques have rapidly advanced the manipulation of small objects [1,2,3,4]. These manipulation techniques can be classified as contact and non-contact types of manipulation. Contact-type manipulation includes various micro- and nano-manipulators, such as glass needles driven by piezoelectric actuators [5], microelectromechanical systems (MEMS) tweezers driven by thermal expansion [6], electrostatic force [7], and magnetic force [8], and nanotweezers using nanoscale probes, such as carbon nanotubes [9]. Conversely, non-contact manipulation techniques manipulate small objects using optical radiation pressure [10], acoustic pressure [11], magnetic field [12], and electric field [13]. Both types of manipulation techniques have been widely used in micro- and nano-technologies and biological research.

As contact-type manipulators grasp objects directly and physically, fewer restrictions exist in terms of materials and sizes of objects that can be grasped compared to those of non-contact-type manipulators. Additionally, contact-type manipulators can conveniently move between different environments, such as air and liquid, owing to the direct contact with the target objects. Furthermore, it can be used for advanced manipulation, such as physical stimulation of living cells [14].

Typically, contact-type manipulators, such as MEMS tweezers, use compliant mechanisms to deform end effectors and grasp target objects. This is because complicated link mechanisms are not suitable for the miniaturization of both end effectors and actuators [6,7,8]. Unlike conventional link mechanisms, the absence of joints in a compliant mechanism renders it with the potential to realize a compact and low-maintenance manipulator. However, micro-manipulators with compliant mechanisms are generally designed using trial-and-error methods based on the designer’s experience [15]. Therefore, it is intrinsically difficult to determine an optimal mechanism to minimize the size of the manipulator and maximize the displacement of end effectors with a small driving force in a short time.

To overcome this problem, topology optimization is considered a promising design method to obtain an optimal mechanism [16]. Topology optimization is a type of structural optimization that provides the highest degree of freedom in the design of compliant mechanisms. Herein, both shape and configurations, such as the number of holes inside a rigid body, can be altered. In this method, the optimal structure for the desired output can be determined by setting boundary conditions, such as the design area, fixed parts, and position and direction of the input and output of the load. Typically, the shape obtained by topology optimization is extremely complex, rendering its fabrication using conventional fabrication processes difficult owing to high design flexibility. However, three-dimensional (3D) printing can be used to fabricate these complex shapes.

In this study, we developed micro-tweezers with a compliant mechanism by combining a topology optimization method and a microstereolithography technique using a blue semiconductor laser, which is a high-precision 3D printing method developed previously [17]. Topology optimization was used based on the level set method [18], and the designed micro-tweezers maximized the displacement of the tweezer tip proportional to the input load based on mechanical evidence. The designed micro-tweezers were fabricated using an in-house microstereolithography system. Additionally, we constructed an actuation system to drive and control the 3D-printed micro-tweezers and demonstrated the manipulation of an organoid with a diameter of approximately 360 µm.

This manuscript contains the following sections: Section 2 covers the topology optimization method, fabrication system and materials for microstereolithography, the actuation system of the micro-tweezers and preparation of biological samples. Section 3 describes the design and experimental verification of the topology-optimized micro-tweezers and demonstration of manipulation of an organoid using the micro-tweezers. Finally, the conclusions are drawn in Section 4.

## 2. Materials and Methods

### 2.1. Topology Optimization of Micro-Tweezers with a Compliant Mechanism

The designed micro-tweezers with a compliant mechanism used topology optimization to enable the grasping of microscale biological samples. Herein, the optimum shape was obtained by maximizing the value of the objective function without relying on trial-and-error results. We used the structural optimization algorithm of the compliant mechanism based on the level set method developed by Yamada et al. [18].

**Figure 1:**
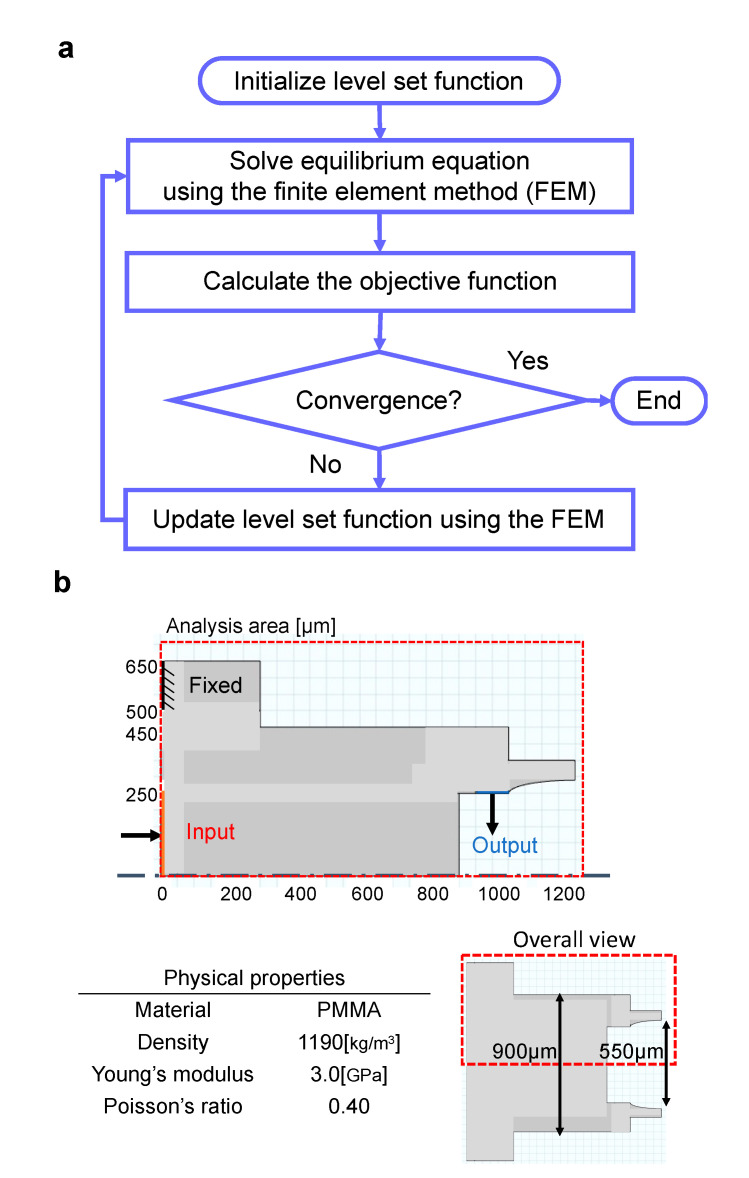
Outline of the topology optimization method and design conditions: (**a**) Flowchart of the algorithm used for topology optimization; (**b**) design conditions and physical properties.

Figure 1a depicts the flowchart of the algorithm. As illustrated in the figure, a level set function representing the proper initial material distribution is predetermined. In the next step, the equilibrium equation is solved using the finite element method (FEM). The third step involves calculating the objective function, wherein the optimization process ends when the objective function converges; otherwise, the sensitivity to the objective function is calculated. Here, we set the objective function to maximize the displacement of the tweezers’ tip with respect to the input load. In the fourth step, the level set function is updated based on the time evolution equation with the boundary conditions using FEM. A commercial Multiphysics software (COMSOL Multiphysics version 5.4, COMSOL, Inc., Burlington, MA, USA) was used as a tool to execute the FEM. Therefore, the optimum shape of tweezers with a compliant mechanism was determined by performing repetitive calculations until the convergence of the objective function was obtained.

Figure 1b depicts the design area and boundary conditions used in the optimization calculation. In this study, the analysis area was considered for manipulating a biological sample (organoid) with a maximum diameter of 550 μm. As illustrated in Figure 1b, the input, output, and fixed parts were set, and the optimization process was performed to maximize the displacement of the tweezer tip with respect to the input load. In this structure, the tip of the tweezers was closed by pressing the input. Although the photocurable resin used for the fabrication was a type of acrylate resin, the analysis was performed by approximating the values of mechanical properties to those of a polymer material, namely polymethyl methacrylate (PMMA).

### 2.2. Microstereolithography System Using a 405-nm Blue Laser

Previously, a bottom-up approach-based microstereolithography system that can fabricate 3D microstructures with a resolution of several micrometers using a 405-nm blue laser was developed to realize the additive fabrication of micro-tweezers on a glass capillary [17]. In this study, this microstereolithography system was used to fabricate micro-tweezers with a compliant mechanism on a glass capillary using topology optimization.

**Figure 2:**
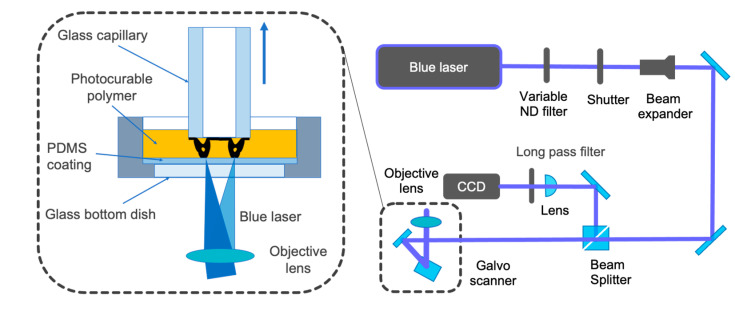
High-resolution microstereolithography system using a blue laser.

Figure 2 depicts a schematic of the proposed fabrication system. The laser beam emitted from a semiconductor laser (06-MLD, Cobolt AB, Solna, Stockholm, Sweden) with a wavelength of 405 nm was passed through a variable neutral density (ND) filter, mechanical shutter, beam expander, and two-axis galvo scanner (GM-1015, Canon Inc., Tokyo, Japan). Subsequently, the laser beam was focused on a photocurable resin using an objective lens (Plan N 4×, NA = 0.1, Olympus Corp., Tokyo, Japan). The galvo scanner scans the focused laser spot in the focal plane to fabricate an arbitrary two-dimensional shape and pulls up the glass capillary layer-by-layer to fabricate an arbitrary 3D shape. This bottom-up approach enables the fabrication of a high-aspect-ratio structure with a height of 8.5 cm by moving the z-stage, wherein the jig for mounting the glass capillary is installed. Additionally, an optical system comprising a green light-emitting diode ring-shaped illuminator, a long-pass filter, an imaging lens, and a charge-coupled device camera is combined with the proposed fabrication system. This entire observation system enabled us to monitor the fabricated structure and the scanning laser beam during fabrication. Furthermore, the observation system aids in aligning the focal spot on the substrate and prevents the peeling and misalignment of fabricated objects. The output laser power was adjusted using a variable ND filter to optimize the fabrication conditions. Furthermore, the scanning speed and trajectory of the laser beam were optimized to fabricate highly precise topology-optimized micro-tweezers.

### 2.3. Preparation of the Photocurable Polymer

We prepared a photocurable resin suitable for high-resolution microstereolithography that uses a 405-nm blue laser. We used a light absorber that strongly absorbs blue light with a wavelength of 405 nm in a photopolymer to achieve high-resolution fabrication [17]. Mixing the light absorber into the photocurable resin increased the absorption rate of the blue laser in the photocurable polymer and decreased the size of the cured voxel. This resin ensures that microscale resolution is achieved without using a femtosecond pulsed laser, which is typically required in two-photon microstereolithography [19]. The prepared photocurable polymer comprises 95.9 wt% acrylate resin (SR499, Sartomer USA LLC., Exton, PA, USA), 1.0 wt% photopolymerization initiator (diphenyl (2,4,6-trimethylbenzoyl) phosphine oxide (TPO), Sigma-Aldrich Co. LLC., St. Louis, MO, USA), 3.0 wt% polymerization inhibitor (2-tert-butyl-4-methylphenol, Sigma-Aldrich Co. LLC., St. Louis, MO, USA), and 0.1 wt% blue light absorber (FDB-009, Yamada Chemical Co., Ltd., Kyoto, Japan). These materials were mixed using a mixer (ARE-250, Thinky Corp., Tokyo, Japan) and degassed at 2000 rpm for 5 min in the mixing and defoaming modes, respectively. Subsequently, the mixture was stirred at 60 rpm for 24 h using a ball mill. The fabricated 3D object was washed using a solvent (Solfit: 3-methoxy-3-methyl-1-butanol, Kuraray Co., Ltd., Tokyo, Japan) and ethanol for 10 min.

### 2.4. Preparation of a Glass-Bottom Dish and Glass Capillary

The photocurable polymer in the proposed microstereolithography was stored in a glass-bottom dish. Typically, the fabricated layer must be peeled off from the surface of the glass-bottom dish, fabricating the next layer in the case of a bottom-up approach. Therefore, polydimethylsiloxane (PDMS), which is an oxygen-permeable resin, was coated on the surface of the glass-bottom dish to prevent adhesion of the photocurable polymer. Additionally, the curing of radical polymerization-type photopolymer is inhibited by the presence of oxygen on the surface of the PDMS film [20], and the fabricated 3D object can be peeled off from the bottom surface of the glass substrate easily. Furthermore, a PDMS prepolymer solution (Silpot 184, Dow Corning Toray Co., Ltd., Tokyo, Japan) comprising a silicone elastomer and a curing agent was mixed at a ratio of 10:1 to generate the PDMS film on the glass-bottom dish; the vacuum generated was degassed. Subsequently, the prepolymer solution was applied on the surface of the glass-bottom dish, spin-coated at 2000 rpm for 10 s, and cured at 60 °C for 3 h.

### 2.5. Actuation System for Micro-Tweezers

**Figure 3:**
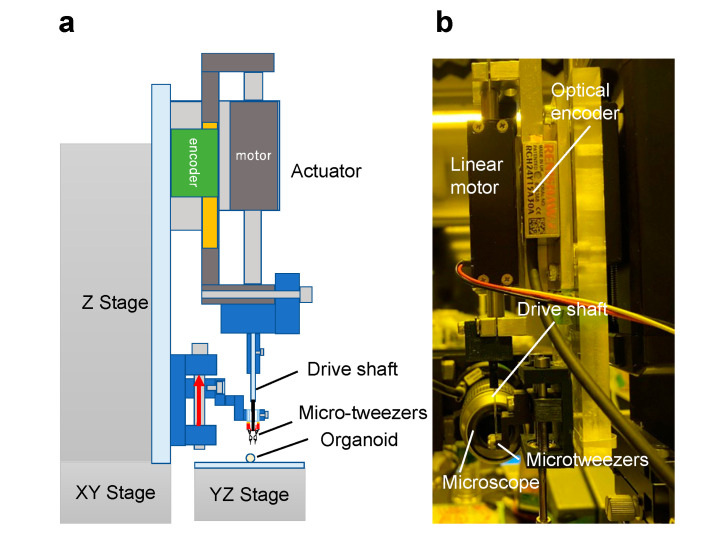
Developed actuation system for micro-tweezers using a linear motor. (**a**) Schematic of the developed system; (**b**) photo of the developed system with microscope for the observation of micro-tweezers’ motion.

To drive the fabricated micro-tweezers, we constructed an actuation system that can apply a load to open and close the tweezers. Figure 3a,b show the schematic and photograph of the actuation system. This system comprises a linear motor (S080Q, GMC HILLSTONE Co., Ltd., Yamagata, Japan), an encoder (RCH24Y15A30A, Renishaw plc., Gloucestershire, UK), and a drive shaft that applies the load to the tweezers. The linear motor attached to the z-stage located above the micro-tweezers pushes it downward. The input load to the tweezers is controlled by force estimation using a linear encoder as a reaction force observer [21,22]. Moreover, the feedback control reduces the influence of environmental disturbances and enables a stable control of forces without the use of force sensors. Furthermore, the tweezer tip was observed under a microscope (VHX-900, Keyence Corp., Osaka, Japan) when manipulating a target sample to discern the motion of micro-tweezers driven by the proposed actuation system.

### 2.6. Preparation of Organoids

To demonstrate the capability of the proposed micro-tweezers in handling biological samples, hair follicle germs were used in the experiment [23]. Mouse epithelial and mesenchymal cells were seeded in a non-cell-adhesive round-bottom 96-well plate (Prime surface 96U, Sumitomo Bakelite Co., Ltd., Tokyo, Japan) at a density of 3 × 10^3^ cells/well of each cell type. The cells were cultured for three days in Dulbecco’s modified Eagle’s medium (Sigma-Aldrich Co. LLC., St. Louis, MO, USA) and keratinocyte growth medium-2 (Kurabo Industries Ltd., Osaka, Japan) at a ratio of 1:1. This culture medium was supplemented with 10% fetal bovine serum (Sigma-Aldrich Co. LLC., St. Louis, MO, USA) and 1% penicillin-streptomycin solution (Thermo Fisher Scientific, Waltham, MA, USA) in a humidified environment at 37 °C and 5% CO_2_. The cultured hair follicle germs were fixed with a 4% paraformaldehyde solution. These hair follicle germs were manipulated using the proposed micro-tweezers in a phosphate-buffered saline (PBS) solution.

## 3. Results and Discussion

### 3.1. Topology Optimization Results and Simulation of the Motion of Micro-Tweezers

**Figure 4:**
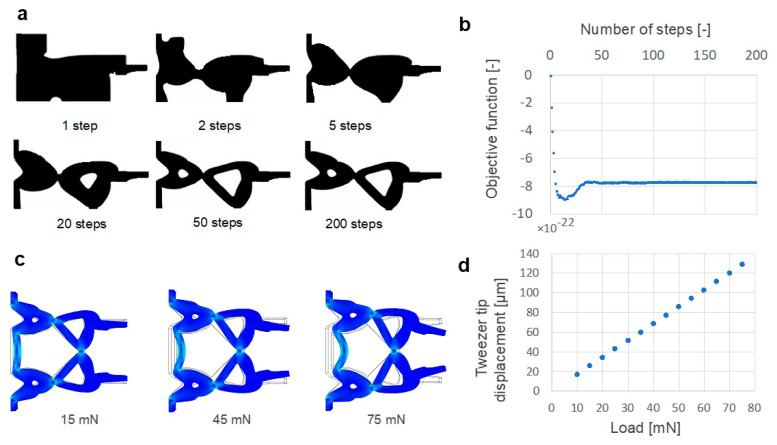
Results of topology optimization and finite element analysis: (**a**) Shape transition based on the number of calculation steps; (**b**) Convergence of the objective function; (**c**) Simulation results of the deformation of tweezers when the load is applied to the input of the tweezers (15 mN, 45 mN, 75 mN); (**d**) Displacement of the tweezer tip.

Topology optimization used to design the micro-tweezers with a compliant mechanism ensured the manipulation of an organoid with a maximum diameter of 550 µm. Figure 4a depicts the convergence of the objective function and shape transition for 1, 5, 10, 20, 50, and 200 steps during repetitive calculations. The results indicate that the regions that did not contribute to maximizing the displacement of the micro-tweezer tip were eliminated with the increasing number of steps. Additionally, structurally flexible parts were generated to realize the compliant mechanism. In this calculation, the convergence of the objective function was verified in approximately 200 steps (Figure 4b). Thus, after duplicating this shape on the axis of symmetry, a computer-aided design model of the topology-optimized micro-tweezers was prepared to perform the finite element analysis (FEA) and fabricate the 3D micro-tweezers.

FEA was used to simulate the operation of the tweezers (COMSOL Multiphysics version 5.4) to confirm the deformation of micro-tweezers when the load was applied to the input. The mechanical properties of a typical PMMA were used in this simulation. The values of Young’s modulus of the tweezers and Poisson’s ratio were set to 3.0 GPa and 0.40, respectively. Figure 4c depicts the von Mises stress and deformation behavior, whereas Figure 4d illustrates the displacement of the tweezer tip as a function of the input load. These results analytically demonstrate that the tweezers close based on the applied load owing to the flexibility of the shape and material. Additionally, we verified that the tip of the tweezers closes linearly when the load is applied, and the displacement of the tip is sufficient to grasp an organoid between the micro-tweezer tips.

### 3.2. Fabrication of Micro-Tweezers for Manipulating an Organoid

**Figure 5:**
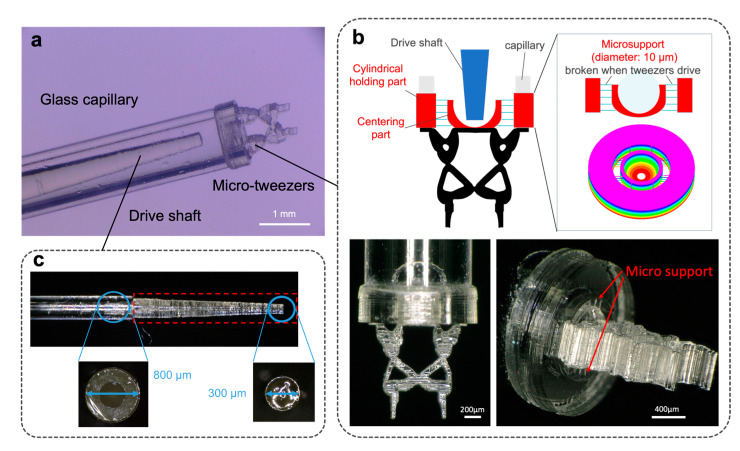
Topology-optimized micro-tweezers fabricated using microstereolithography: (**a**) Overall view of the fabricated micro-tweezers on a glass capillary with a drive shaft; (**b**) Details of the base parts to support the tweezers on the glass capillary; (**c**) Additively fabricated drive shaft used for pushing the tweezers.

The topology-optimized micro-tweezers with a compliant mechanism were additively fabricated on a capillary of inner and outer diameters of 920 and 1390 µm, respectively, using the microstereolithography system (Figure 2). Figure 5a depicts the overall view of the fabricated micro-tweezers on a glass capillary with a drive shaft. Micro-tweezers are located on the tip of the glass capillary, and the drive shaft is placed inside the capillary to push the bottom part of the tweezers. To fabricate the micro-tweezers on the end face of the capillary, we constructed a base with microsupports (diameter = 10 µm) between the centering part and the cylindrical holding part (Figure 5b). The base ensures that the micro-tweezers remain intact at the center of the capillary and adhere to the end face of the capillary. The bowl-shaped centering part centers the drive shaft to push the micro-tweezers vertically. The microsupports can be broken and separated from the system by pushing the centering part using the drive shaft. Consequently, the micro-tweezers can be operated without any constraining force or friction. Additionally, a tapered column with a tip diameter of 300 µm was fabricated on the glass capillary (outer diameter = 800 µm) to push the centering part through the outer glass capillary (Figure 5c). The tapered column ensures that the tip of the drive shaft encounters only the centering part of the tweezers.

### 3.3. Manipulation of an Organoid Using the Topology-Optimized Micro-Tweezers

**Figure 6:**
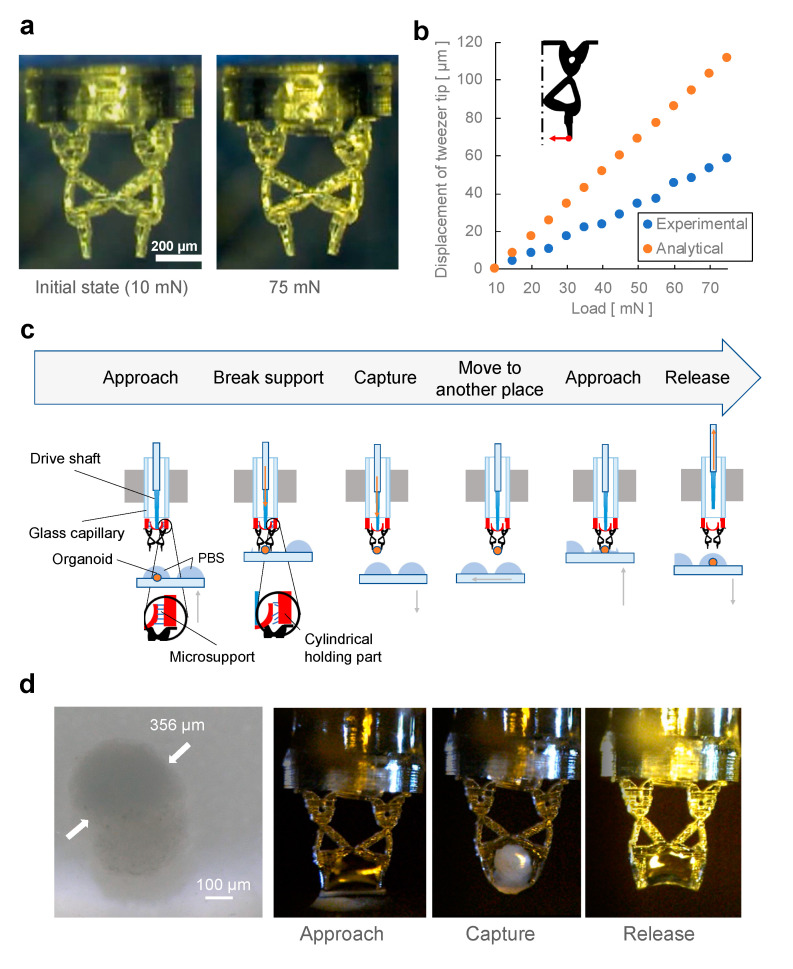
Evaluation of micro-tweezers and demonstration of capture and release of an organoid: (**a**) actuation of micro-tweezers with different applied loads; (**b**) comparison of simulation and experimental results of the deformation of the tweezer tip under the applied load (10 mN to 75 mN); (**c**) procedure of capturing an organoid; (**d**) optical microscopic images of an organoid and its manipulation in phosphate-buffered saline solution.

Initially, we validated the motion behavior of the micro-tweezers fabricated using microstereolithography. Figure 6a depicts the state of the tweezers with and without the applied load. The comparison of these microscopic optical images of the tweezers demonstrates that sufficient displacement occurred for the tweezers to grasp an organoid with a diameter of approximately 360 µm when the load was applied. Figure 6b compares the displacement of the tweezer tip in the FEA and an experiment using the tweezers, considering an applied load of 10 mN to 75 mN. Herein, the initial position of the tweezers is considered when a load of 10 mN is applied as the drive shaft may be off-center before pushing. Based on this comparison, the displacement of the tweezer tip was proportional to the applied load in both the FEA and experiment. However, the tweezers fabricated using microstereolithography required a larger load to obtain the displacement identical to that of the analytical result. This can be attributed to the shape of the fabricated tweezers, which was larger than the designed shape owing to the excess exposure of laser irradiation during the fabrication process. For example, at the thinnest part in the center of the micro-tweezers, the width was approximately 5 µm larger than the designed width. Additionally, the mechanical properties of the material of the tweezers (acrylate resin) used in the experiment may differ from those of the PMMA used in the analysis. The mechanical properties of polymer materials, such as Young’s modulus, vary depending on the purity of the material, the presence of additives, and the polymerization conditions and so on. There is the possibility that the Young’s modulus of the actually fabricated material was higher than that of the material library of the analysis software. In the future, it is expected that the analytical results will match the experimental results by using the actual measured values of the physical properties of the photocurable resin used to make the tweezers.

Durability of the tweezers is an important issue for the practical use of such micro-actuators. It was confirmed that the displacement of the tweezer tip was almost constant for at least 50 repetitions of open and close motions between the load of 10 mN to 25 mN in the experiments. For further improvement of durability, it is necessary to reduce excessive stress concentration by minimizing the steps that occur on the sides of 3D objects due to the accumulation process. For example, a smaller accumulation pitch or continuous upward pulling of the base substrate is effective to reduce the accumulation steps. Additionally, it will be possible to fabricate durable tweezers using the topology optimization with multiple materials of different mechanical stiffness, where soft materials are placed only in high-stress areas. Such tweezers of multi-material constitution will be produced by using a multi-material microstereolithography [24].

Furthermore, we demonstrated the manipulation of an organoid in PBS using the proposed micro-tweezers. Figure 6c illustrates the organoid manipulation procedure. Initially, the tweezers approached the organoid placed in PBS solution, and the load was applied to the centering part of the tweezers using the drive shaft to break the microsupports. Subsequently, the drive shaft pushed further and deformed the tweezers, which grasped the organoid. The tweezer was moved to another position to release the organoid by removing the load applied to the drive shaft. Figure 6d depicts the results of the organoid manipulation. The organoid was fixed with formaldehyde and was gripped on the side that measured approximately 360 µm. These sequential images demonstrate that the organoids in PBS were grasped, transported, and released with stable gripping without breaking the tweezers. The load on the drive shaft was 60 mN when the organoid was grasped in the experiment. These results verify that small and soft biological samples can be successfully manipulated using 3D-printed topology-optimized micro-tweezers with a compliant mechanism.

## 4. Conclusions

To manipulate organoids effectively, we developed topology-optimized micro-tweezers with a compliant mechanism, fabricated by high-resolution microstereolithography using a blue laser. An actuation system was constructed with force control to operate the proposed micro-tweezers, and its motion was analytically and experimentally evaluated. The results confirmed that the displacement of the tweezer tip was proportional to the applied load. Furthermore, we successfully demonstrated the manipulation of an organoid with a diameter of approximately 360 µm. In the current actuation system, the targeted organoid was observed using optical microscope images and the position of the micro-tweezers was manually adjusted to the sample, so the tweezers were cumbersome to operate. To overcome the problem, it is preferable to introduce position control using microscope images. In addition, although topology optimization was performed in two dimensions, microstereolithography can be used to fabricate complex 3D structures. Therefore, it is expected that 3D micro-tweezers will be fabricated by adopting topology optimization in three dimensions. In the future, the additive manufacturing of topology-optimized micro-tweezers can potentially offer customized microtools for the manipulation of various types of microscale objects and biological samples, such as cells, spheroids, and organoids.
